# Individual behaviour, growth, survival and vulnerability to hunting in a large mammal

**DOI:** 10.1002/ece3.11003

**Published:** 2024-02-13

**Authors:** Pier‐Oliver Cusson, Fanie Pelletier

**Affiliations:** ^1^ Département de Biologie Université de Sherbrooke Sherbrooke Québec Canada

**Keywords:** animal personality, bighorn sheep, exploitation, harvest‐induced selection, hunting vulnerability, pace‐of‐life

## Abstract

Humans have exploited wild animals for thousands of years. Recent studies indicate that harvest‐induced selection on life‐history and morphological traits may lead to ecological and evolutionary changes. Less attention has been given to harvest‐induced selection on behavioural traits, especially in terrestrial systems. We assessed in a wild population of large terrestrial mammals whether decades of hunting led to harvest‐induced selection on trappability, a proxy of risk‐taking behaviour. We investigated links between trappability, horn growth and survival across individuals in early life and quantified the correlations between early‐life trappability and horn growth with availability to hunters and probability of being shot. We found positive among‐individual correlations between early‐life trappability and horn growth, early‐life trappability and survival and early‐life horn growth and survival. Faster growing individuals were more likely to be available to hunters and shot at a young age. We found no correlations between early‐life trappability and availability to hunters or probability of being shot. Our results show that correlations between behaviour and growth can occur in wild terrestrial population but may be context dependent. This result highlights the difficulty in formulating general predictions about harvest‐induced selection on behaviour, which can be affected by species ecology, harvesting regulations and harvesting methods used. Future studies should investigate mechanisms linking physiological, behavioural and morphological traits and how this effects harvest vulnerability to evaluate the potential for harvest to drive selection on behaviour in wild animal populations.

## Graphical abstract

We quantified among‐individual correlations in behaviour, growth and survival in a wild bighorn sheep population and assessed whether decades of size‐selective hunting led to indirect harvest‐induced selection on behaviour. We found positive among‐individual correlations between early‐life trappability, growth and survival but no correlations between early‐life behaviour and vulnerability to hunters. Our results show that correlations between behaviour, growth and life‐history traits can occur in wild terrestrial population but may be context dependent and highlight the difficulty in formulating general predictions about harvest‐induced selection on behaviour.

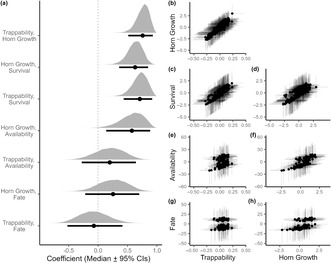

## 1 INTRODUCTION

Humans have exploited wild animals for thousands of years (Sullivan et al., 2017). Recently, research has revealed that intense selective removal of individuals based on specific phenotypic traits could cause a selective pressure (Darimont et al., 2015), leading to rapid phenotypic responses in harvested populations (Darimont et al., 2009) that are sometimes associated with genetic changes (Allendorf et al., 2008; Jorgensen et al., 2007; Pigeon et al., 2016). Responses to harvest‐induced selection have been documented for morphological and life‐history traits. For example, in commercial fisheries where large fish are typically targeted, studies have reported reduced size and age at maturation (Jorgensen et al., 2007; Sharpe & Hendry, 2009). In terrestrial systems, selective removal of fast‐growing male bighorn sheep (*Ovis canadensis*) through trophy hunting induced an evolutionary response for reduced horn length (Coltman et al., 2003; Pigeon et al., 2016).

Less attention has been given to the effects of harvest‐induced selection on behavioural traits, especially in terrestrial systems (Leclerc et al., 2017). Yet, differences in behaviour can affect the likelihood of individuals being harvested (Biro, 2013; Biro & Dingemanse, 2009; but see Jolly et al., 2019) and these differences are often heritable (Dochtermann et al., 2015). For example, in fisheries, passive gears such as angling, gill netting and trapping have been shown to selectively remove bolder, risk‐prone individuals (e.g. Biro & Post, 2008; Diaz Pauli et al., 2015; but see Wilson et al., 2011). As a result, many exploited populations may experience a decrease in boldness due to plastic or evolutionary changes in the distribution of behaviour (Arlinghaus et al., 2017, 2016). Harvest‐induced selection on behaviour can have population, community and even ecosystem‐wide ecological consequences impacting harvest potential (Arlinghaus et al., 2017; Diaz Pauli & Sih, 2017).

Behavioural and life‐history traits can be correlated (Biro & Stamps, 2008; Réale et al., 2000, 2009) and some authors have suggested that they form an integrated phenotype (Montiglio et al., 2018; Réale et al., 2010), but empirical support for this hypothesis has been mixed (Royauté et al., 2018). Among life‐history traits, growth rate, in particular, seems to be linked to behavioural traits (Royauté et al., 2018). Bolder, more aggressive and active individuals appear to consistently have higher food intake and growth rate than shy individuals (Biro & Stamps, 2008), even when food is unlimited (Biro et al., 2014). Such associations between life‐history and behavioural traits can further increase the ecological and practical implications of selective harvesting. Size‐selective harvest methods or regulations can, for example, induce a selective pressure on behavioural traits because bolder, more active individuals might reach larger size faster and thus be more vulnerable to harvesting. Experimental selective removal of large individuals has indeed resulted in declines in consumption rates and willingness to forage under predation threat in Atlantic silverside (*Menidia menidia*) (Walsh et al., 2006) and reduced exploration and boldness in zebrafish (*Danio rerio*) (Uusi‐Heikkilä et al., 2015). Hence, the common management strategy of imposing a minimal size for harvest could lead to population‐level changes in morphological, life‐history and behavioural traits in exploited populations.

Harvest of wild terrestrial population through hunting can also lead to selection (Festa‐Bianchet, 2003). Hunting can induce life‐history and morphological changes such as prolonged maternal care (Van de Walle et al., 2018), earlier birth dates (Gamelon et al., 2011) and reduced male weapon size (Coltman et al., 2003; Pigeon et al., 2016). Terrestrial animals also exhibit consistent individual differences in behaviour (Réale et al., 2000) and hunting can act both directly or indirectly as a selective pressure on behavioural traits (Leclerc et al., 2017, 2019). Wapiti (*Cervus canadensis*) and European red deer (*Cervus elaphus*) that were bolder and more active during the hunting season were, for example, at greater risk of being shot (Ciuti et al., 2012; Lone et al., 2015). Conversely, in a Swedish population of brown bears (*Ursus arctos*) where most hunters use dogs to find and track bears, more active bears were more likely to survive the hunting season because they may be better at escaping dogs (Leclerc et al., 2019). These studies reported direct selection on behaviour but did not assess the links between individual growth rate or size and behaviour, which could lead to additional indirect selective effects of hunting. Indeed, if bolder or more active individuals are also fast‐growing and/or larger, biases in removal for certain behavioural phenotype could lead to indirect selection on growth rate or size (Biro & Post, 2008). Similarly, if bolder individuals take more risks to obtain food resources and achieve higher growth rates, size selective hunting could lead to indirect selection against bolder risk‐taking individuals (Sasaki et al., 2009).

Here, we use data from a long‐term monitoring programme of a wild population of bighorn sheep that were subjected to trophy hunting for decades to investigate whether size‐selective hunting also selects trappability, a proxy of risk taking. We chose a risk‐taking behaviour because it can affect both the growth rate (Biro & Stamps, 2008) and survival (Ciuti et al., 2012) of individuals and was previously studied in this population (Réale et al., 2009, 2000; Réale & Festa‐Bianchet, 2003). Using multivariate analyses, we evaluated whether individuals exhibited consistent individual differences in trappability and growth. We tested for among‐individuals correlations between early‐life trappability, growth and survival. We also tested how early‐life trappability and growth related to hunting vulnerability later in life by looking at their correlations with two factors: availability to hunters and probability of being shot (hereafter referenced as fate) before the age of potential high reproductive success. As reported in some aquatic systems, we expected that individuals would consistently differ in behaviour and growth, and that risk taking should be positively correlated with growth. Because trophy hunting is by regulation size‐selective (Festa‐Bianchet et al., 2014), we hypothesised that a positive link between trappability and horn growth would lead to hunting‐induced selection on behaviour. We thus expected trappability behaviour to increase the probability of individuals being available for and shot by hunters.

## 2 MATERIALS AND METHODS

### 2.1 Study system and data collection

The Ram Mountain bighorn sheep population is on a mountainous outcrop in Alberta, Canada, 30 km east of the Rockies (52°N, 115°W, elevation 1080–2170 m). The study area consists of 38 km^2^ of alpine and subalpine habitat including rock scree, cliffs and alpine meadows. Monitoring of this population has been ongoing since 1971 (Jorgenson et al., 1993). Sheep were marked with ear tags or collars and captured each year from late May to late September in a corral trap baited with salt. Most individuals (95%) were first captured as lambs or yearlings. For sheep caught as adults, age was determined using horn annuli (Geist, 1966). Marked sheep were monitored for their entire lifetime. The annual resighting rate was high (95% for males, 99% for females) and emigration rare (Jorgenson et al., 1997). Individuals not seen in the study area for two consecutive years were therefore considered dead and we used the year of last sighting as their last year of life. Causes of death were rarely known except for hunted males, because hunters were required to register their kill.

The study population was subjected to trophy hunting until 2011. From late August to October ‘legal’ males were at risk of being shot. The definition of ‘legal’ male was based on minimum horn curl, which is correlated with horn length (Festa‐Bianchet et al., 2014). The degree of horn curl necessary to be deemed ‘legal’ was 4/5 until 1996, when it was changed to full curl, a more restrictive regulation (see figure S1 in Pigeon et al., 2016). Under the 4/5 horn curl regulation, a mean of 2.26 males were shot each year. When the regulation changed to full curl in 1996, the harvest rate dropped to 0.27 males per year until hunting was closed in 2011. As in Pigeon et al. (2016), we therefore considered the trophy hunting period to be prior to 1996 (see also Pelletier & Coltman, 2018). The annual harvest rate of ‘legal’ males was about 40% (Coltman et al., 2003). Rams with small horns were always protected from hunting.

Bighorn sheep horns stop growing during winter, creating rings or annuli, which clearly define each annual horn increment. We measured the length of these increments to the nearest mm for males aged 1 to 4 years to obtain an age‐specific measure of growth. This corresponds to increments 2 to 5, which account for more than 75% of asymptotic horn length of mature males (Bonenfant et al., 2009). When data on both horns were available, we used the longer of the two measurements. Missing values occurred when individuals died over winter at age 4 or younger so horn growth in the previous summer could not be measured using annuli length. In these cases, we measured horn growth as the difference between horn length in September and horn length in September of the previous year. Horn lengths were adjusted to September 15 each year using a mixed model approach based on individual recaptures (Martin & Pelletier, 2011). A total of 732 measures of horn growth were available, 649 from annuli length and 83 from the difference in horn length, from 245 unique individuals (mean = 2.98 measures per individual, standard deviation = 1.22, range = 1 to 4).

We used the number of captures, that is, trappability, in a field season as a measure of risk taking, a behaviour which affects sheep survival in risky environments (Réale & Festa‐Bianchet, 2003). As in previous studies on this population, we assumed that number of captures reflected the level of acceptance of the risk involved in licking salt in the trap (see Réale et al., 2000 for justifications). Highly trappable individuals were therefore considered to be more risk‐prone, while rarely captured males were considered less risk‐prone (Réale et al., 2000, 2009). The trap was open daily each year from late May to late September. Captures occurred when a group of sheep entered the trap. Much of the variability in the number of captures was due to how willing individuals are to enter the trap once they are next to it (Réale et al., 2000). The same trap has been used since the beginning of the project, in the same location (see Poissant et al., 2013). It is located on an alpine meadow and individuals can walk all around the trap. Number of captures refers to the number of times an individual was trapped and handled. That number is often lower than the number of times a male was actually caught in the trap because those trapped within 21 days of being handled were released without handling and their capture was not noted. The annual number of captures thus followed an underdispersed Poisson distribution with a range of 0–7 captures.

We excluded data on lambs because the first horn increment wears out rapidly (Geist, 1966) and because their capture was not independent of their mother's behaviour, as lambs often entered the trap following their mother. We also omitted data on eight immigrant males because they did not face the same early‐life conditions nor had the same experience of the trap as resident sheep. Immigrants were also not socially integrated to the local population when they first arrived (Poirier & Festa‐Bianchet, 2018). We were left with 813 measures of annual number of captures from 271 unique individuals.

### 2.2 Statistical analysis

We used multivariate analyses to investigate the correlations between early‐life trappability, horn growth, pre‐harvest survival and vulnerability to hunting. As previously described, trappability was measured as the annual number of captures and horn growth was the annual growth in horn length of rams. Survival was fitted as a binomial variable representing the annual survival of a ram. Annual trappability, horn growth and survival were considered from ages 1 to 4. No individuals reached legality before 4 years of age. Hence, we used ages 1 to 4 as the pre‐harvest period.

Males legal before or at age 6 are large, fast‐growing males who can potentially have high reproductive success if they survive to age 7 or older, as rams younger than 7 years generally cannot achieve high dominance status (Coltman et al., 2002), but are at risk of being shot at an early age (Coltman et al., 2003; Pigeon et al., 2016). Thus, hunting vulnerability was defined by two variables: the availability of a ram to hunters and its fate. Availability to hunters was defined by the legality status of the individual. If a ram was legal to harvest based on 4/5 regulation at or prior to 6 years of age, it was assigned a value of 1 (*n* = 61). If a ram had not achieved a 4/5 horn curl by age 6 it was coded 0 as it was not available for hunters (*n* = 55). Males that died before reaching 6 years of age while not being legal were assigned NA (*n* = 155). Fate was defined as whether a ram was shot at or before the age of 6. Males shot when 6 years old or younger were assigned the value 1 (*n* = 29). Individuals that died from natural causes or were shot at later ages were given the value 0 (*n* = 55). Males that died of natural causes before reaching 6 years of age or were never exposed to hunters, as hunting stopped in 1996, were assigned NA for fate (*n* = 187).

We fitted a Bayesian multivariate model using these five traits as response variables. For most of the year, including during our field season, bighorn sheep are segregated between nursery groups, consisting of adult females, lambs and sub‐adult males and bachelor groups consisting of adult males (Geist, 1971). As they age, males gradually transition from nursery groups to bachelor groups. The transition generally happens around 3 years of age, but 2‐ and 3‐year‐old males can switch between nursery and bachelor groups (Festa‐Bianchet, 1991; Ruckstuhl, 1998). Bachelor groups spend more time lying and move less than nursery groups (Ruckstuhl, 1998). In our study system, males in bachelor groups tend to use portions of the study area that are further away from the trap As a result, the annual number of captures tends to decrease with male age. We thus included age as a population‐level effect on trappability. Population size varied between 40 and 232 individuals across the study period. Trapping effort per individual thus varied and was higher when the population was at very low density (<100 individuals), decreasing as population size increased. To control for this in our models, we also included the natural logarithm of population size as a population level effect on trappability.

Age and population density reduce early‐life horn growth of males in the study population (Douhard et al., 2017; Jorgenson et al., 1998). We thus included age and population size as population‐level effects on horn growth. Similar to Jorgenson et al. (1998), age was included as a second‐order polynomial. The first horn increment (lamb growth) is usually the shortest in early life, while the second one is usually the longest followed by a slow decrease in length in the following years (Douhard et al., 2017; Appendix 2). As we excluded the first horn increment, the pattern better fitting early horn growth was a second‐order polynomial.

A previous study of males in this population found that early‐life survival was highest when males were aged 2–3 years old, followed by yearlings and then 4–6 years old (Jorgenson et al., 1997). Thus, we included age as a second order polynomial population‐level effect on pre‐harvest survival. Although density effects on survival were not detected previously (Jorgenson et al., 1997), we also included an effect of population size on pre‐harvest survival as our analyses include a longer time series (23 additional years).

For availability to hunters and hunting fate, we included the average population size between 1 and 4 years old as the only population‐level variable. This controls for differences in early‐life environmental conditions that could have affected early‐life horn growth and potentially availability to hunters and hunting fate.

We included individual identity as a random intercept for each of the five response variables because we were interested in among‐individual differences. Year was as also included as a random intercept for trappability, horn growth and pre‐harvest survival to reflect additional inter‐annual differences not accounted for by age and population size. For availability to hunters and hunting fate, we included a random intercept of year of birth (cohort) instead. Trappability was fitted using a Conway–Maxwell–Poisson distribution to account for underdispersion in the number of captures (Shmueli et al., 2005). Horn growth was modelled following a Gaussian distribution. Pre‐harvest survival, availability to hunters and hunting fate were all binomial variables with a single trial and were thus fitted with a Bernoulli error distribution. We used weakly informative priors for all parameters. Model validation was done by looking at trace plots, doing posterior predictive checks and comparing prior and posterior distributions.

We then calculated the coefficients of among‐individual variation as well as repeatability and adjusted repeatability of trappability and horn growth. Coefficient of among‐individual variation refers to the ratio of the among‐individuals standard deviation to the mean trait value of the population (Holtmann et al., 2017). Repeatability was the proportion of total variance explained by among‐individual variance (i.e. among‐individual variance/total variance). Adjusted repeatability was the proportion of variance not related to fixed effects explained by the among‐individual variance (i.e. among‐individual variance/total variance – fixed effect variance) (Nakagawa & Schielzeth, 2010). We used the posterior distribution of each parameter to calculate the coefficients of variation and repeatabilities following (O'Dea et al., 2021, 2022). In doing so, we obtained distributions of coefficient of variation and repeatability estimates. We also included a correlation term for the random intercept of individual identity for each response variable in the model. We thus obtained posterior distributions of estimates of correlations between early‐life trappability, horn growth and survival prior to hunting as well as early‐life trappability and horn growth with availability to hunters and hunting fate at 6 years of age. All analyses were implemented in R 4.2.1 (R Core Team, 2022). Models were run using the ‘*brms*’ package (Bürkner, 2017, 2018).

## 3 RESULTS

### 3.1 Population‐level effects

**FIGURE 1:**
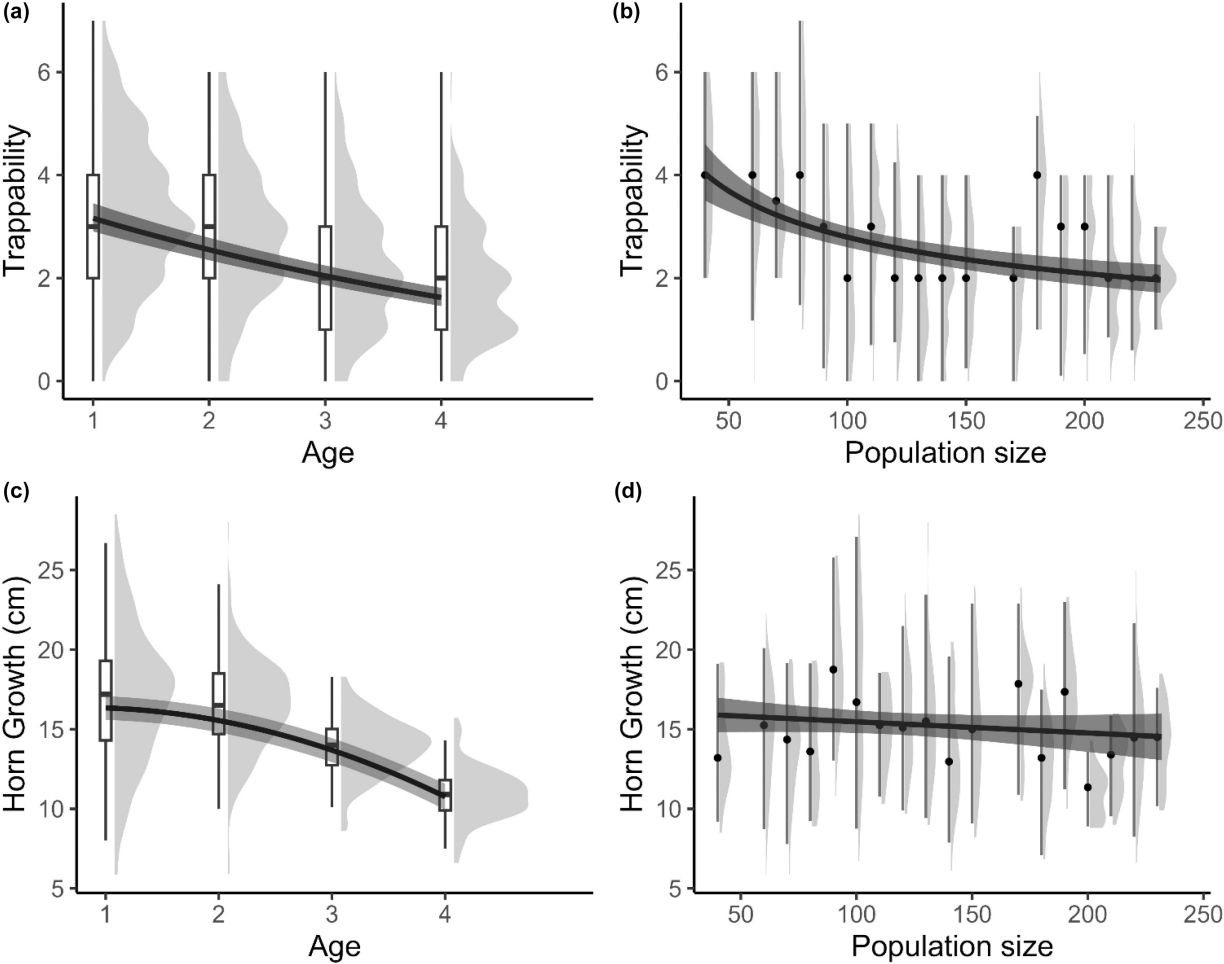
Population‐level effects of age and population size on bighorn sheep rams' trappability (a, b) and horn growth (c, d), at Ram Mountain, Alberta, Canada. The lines represent the model's predicted values of the responses for a given predictor when other predictors in the model are set to their mean value. The shaded areas around the lines represent the 95% uncertainty interval of the predicted responses. The distributions of the raw data are represented in the background, with the median indicated by the dots or the lines at the centre of the boxplots. Population size was grouped by tens to generate the raw data distributions.

The trappability of rams, estimated by the number of yearly captures, declined with increasing age and population size (Table 1, Figure 1a,b). Horn growth also decreased with age, but there was no clear effect of population size on growth (Table 1, Figure 1c,d). Annual survival was not affected by age nor population size (Table 1). Similarly, mean early‐life population size had no effect on availability to hunters and hunting fate at 6 years old or younger (Table 1).

**TABLE 1:** Parameter estimates of the multivariate Bayesian mixed model describing early‐life trappability, horn growth (in cm) and survival, as well as availability to hunters and hunting fate at or prior to 6 years of age at Ram Mountain, Alberta, Canada.

	Trappability	Horn growth	Survival	Availability	Fate
	Estimate (CI_95%_)	Estimate (CI_95%_)	Estimate (CI_95%_)	Estimate (CI_95%_)	Estimate (CI_95%_)
**Population‐Level Effects**					
Intercept	3.23 (2.69 to 3.77)	14.51 (13.78 to 15.23)	1.78 (1.45 to 2.17)	1.18 (−5.32 to 8.26)	−3.24 (−10.93 to 2.62)
Age	−0.19 (−0.21 to −0.17)	−56.07 (−61.25 to −50.80)	−0.67 (−2.57 to 1.23)	–	–
Age^2^	–	−14.93 (−19.82 to −10.07)	−0.48 (−2.36 to 1.37)	–	–
Pop. size	–	−0.42 (−1.08 to 0.22)	−0.11 (−0.46 to 0.20)	–	–
Log (Pop. size)	−0.36 (−0.47 to −0.24)	–	–	–	–
Mean pop. size	–	–	–	–0.24 (−2.02 to 1.56)	−0.58 (−2.43 to 1.32)
**Group‐level effects**					
*~Id (Number of levels: 271)*					
sd(Intercept)	0.15 (0.11 to 0.19)	1.38 (1.05 to 1.73)	1.46 (0.90 to 2.11)	13.79 (5.23 to 25.70)	13.89 (4.42 to 27.32)
*~Year (Number of levels: 43)*					
sd(Intercept)	0.17 (0.13 to 0.23)	1.92 (1.46 to 2.51)	0.44 (0.04 to 0.96)	–	–
*~Cohort (Number of levels: 41)*					
sd(Intercept)	–	–	–	9.85 (2.99 to 20.07)	5.69 (0.30 to 15.32)
*~Observation (Number of levels: 813)*					
sd(Intercept)	0.03 (0.00 to 0.09)	–	–	–	–

*Note*: Estimates are the mean estimate of the posterior distributions and are accompanied by their 95% credibility intervals.

### 3.2 Coefficient of variation and repeatability

**FIGURE 2:**
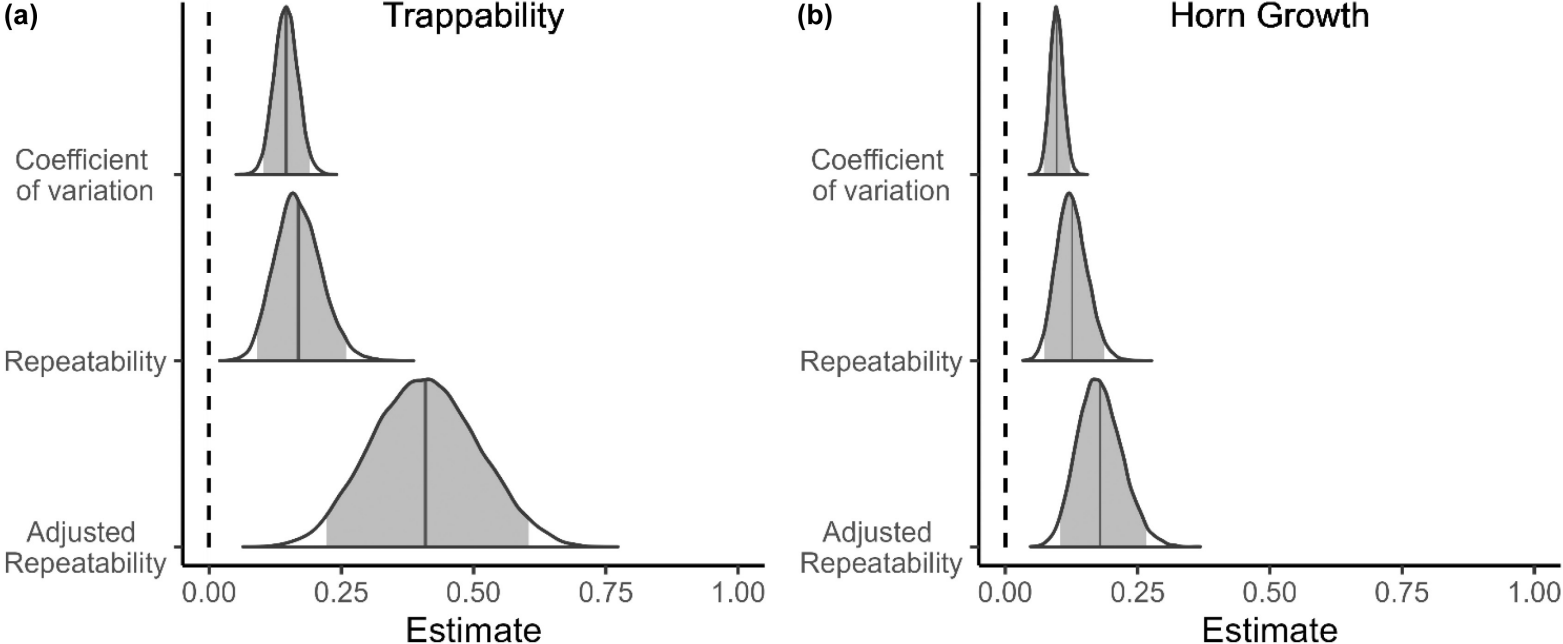
Among‐individual differences in early‐life trappability (a) and horn growth (b) for bighorn sheep rams at Ram Mountain, Alberta, Canada. Repeatability estimates include variation due to fixed effects in the denominator while it was removed for adjusted repeatability estimates. Complete posterior distributions of estimates are illustrated. Shaded areas within the distributions represent the 95% credible intervals. Vertical lines represent the mean estimate.

**FIGURE A1:**
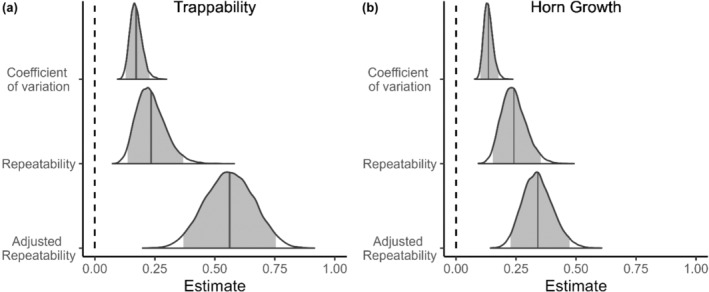
Among‐year differences in early‐life trappability (a) and horn growth (b) for bighorn sheep rams at Ram Mountain, Alberta, Canada. Repeatability estimates include variation due to fixed effects in the denominator while it was removed for adjusted repeatability estimates. Complete posterior distributions of estimates are illustrated. Shaded areas within the distributions represent the 95% credible intervals. Vertical lines represent the mean estimate.

**FIGURE A2:**
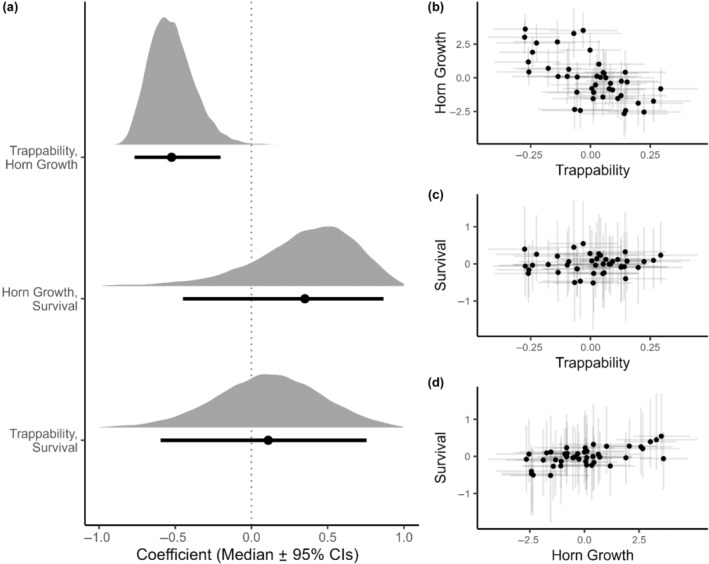
Among‐year correlations between early‐life trappability, horn growth and survival for bighorn sheep rams at Ram Mountain, Alberta, Canada. Complete posterior distributions of estimates are illustrated whit horizontal lines representing the 95% credible intervals and dots representing the mean estimates (a). Relationships between the posterior modes of the ‘Id’ random effect, that is, best linear unbiased predictions (BLUPs), are represented for each correlation for visualisation purposes (b–d).

We found consistent among‐individual differences in early age trappability and horn growth of rams. Mean repeatability estimates (*Rp*) were .169 and .126 for trappability and horn growth, while mean adjusted repeatability estimates (*Rp*_adj_) were, respectively, .409 and .179. Coefficients of variation were .146 for trappability and .097 for horn growth, indicating that the greatest magnitude of differences relative to the population mean was for trappability (see CV in Table 2). In all cases, the 95% credibility intervals of the estimates did not overlap zero (Table 2; Figure 2). Year explained a similar portion of the variance in trappability than sheep identity. Among‐year repeatabilities and coefficient of variation estimates were comparable to the among‐individual estimates. For horn growth, year explained a slightly greater portion of the variance than sheep identity did. The among‐year repeatabilities and coefficient of variation estimates were also slightly higher than the among‐individual estimates (see Appendix 1: Figures A1, A2 and Tables A1, A2).

**TABLE 2:** Among‐individual differences in early‐life trappability and horn growth of bighorn sheep rams at Ram Mountain, Alberta, Canada.

	Trappability	Horn growth
	Estimate (CI_95%_)	Estimate (CI_95%_)
*Rp*	0.169 (0.091–0.259)	0.126 (0.074–0.187)
*Rp*_adj_	0.409 (0.222–0.604)	0.179 (0.104–0.267)
CV	0.146 (0.103–0.190)	0.097 (0.073–0.123)

*Note*: Estimates are the mean estimate of the posterior distributions and are accompanied by their 95% credibility intervals. *Rp* is the repeatability, *Rp*_adj_ is the adjusted repeatability (fixed effect variance removed from denominator) and CV is the coefficient of variation.

### 3.3 Correlations

**FIGURE 3:**
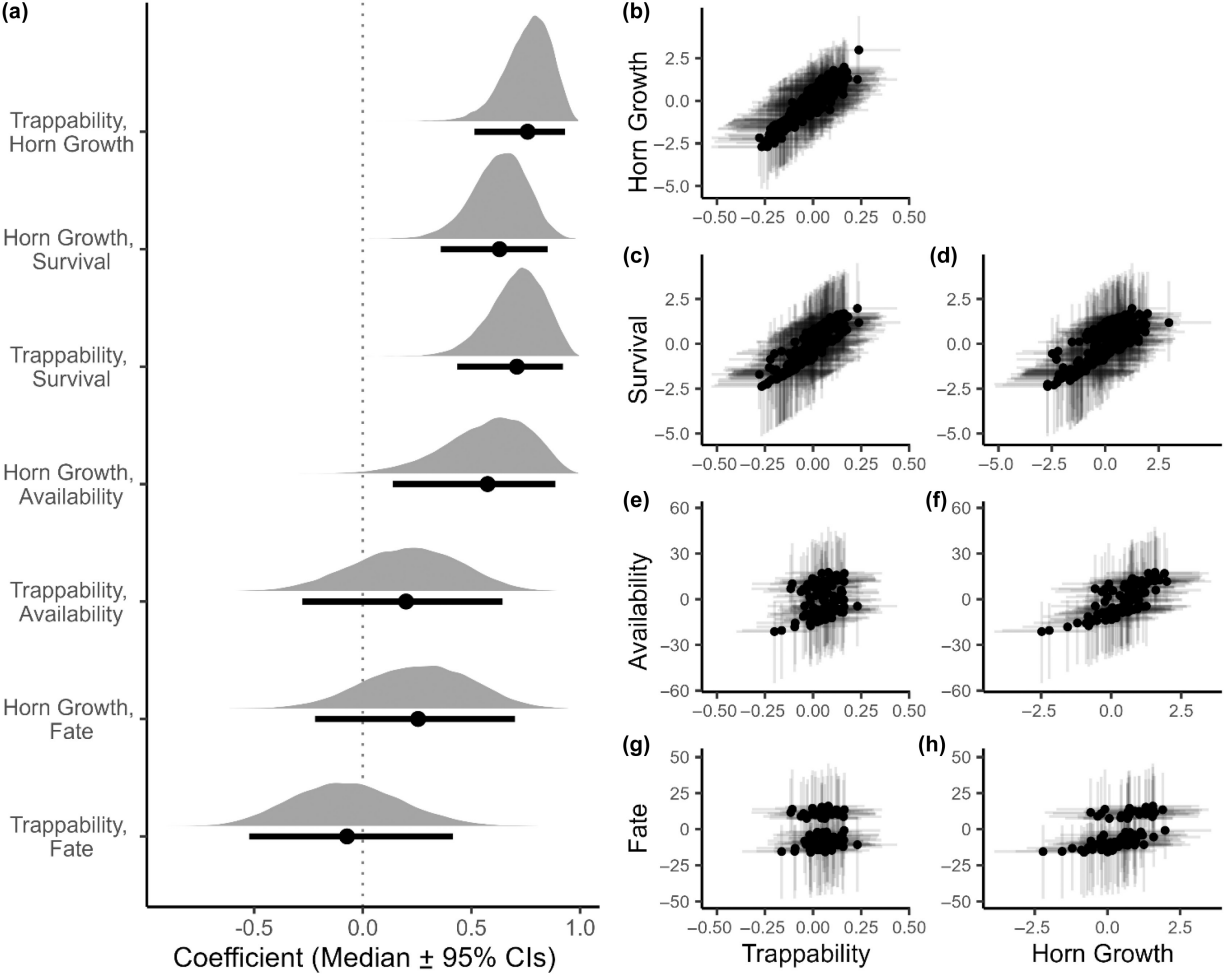
Among‐individual correlations between early‐life trappability, horn growth and survival and availability to hunters and hunting fate at or prior to 6 years of age, for bighorn sheep rams at Ram Mountain, Alberta, Canada. Complete posterior distributions of estimates are illustrated whit horizontal lines representing the 95% credible intervals and dots representing the median estimates (a). Relationships between the posterior modes of the ‘Id’ random effect, that is, best linear unbiased predictions (BLUPs), are represented for each correlation for visualisation purposes (b–h).

We found positive among‐individual correlations between early‐life trappability, horn growth and survival, meaning that more trappable (i.e. risk‐prone) individuals also had on average greater horn growth and higher survival probability (Table 3, Figure 3a–d). We found no correlations between early‐life trappability and availability to hunters or hunting fate at or prior to 6 years among rams that survived to at least 4 years of age (Table 3, Figure 3a,e,g). We did, however, find positive correlations between early‐life horn growth and availability to hunters and hunting fate, although uncertainty around the estimates was higher and the 95% credible interval slightly overlap 0 for the horn growth and hunting fate correlation (Table 3, Figure 3a,f,h). We also found a negative among‐year correlation between horn growth and trappability, but no correlation between among‐year trappability or horn growth and survival (see Appendix 1).

**TABLE 3:** Among‐individual correlations between early‐life trappability, horn growth and survival and availability to hunters and hunting fate at or prior to 6 years of age, of bighorn sheep rams at Ram Mountain, Alberta, Canada.

	Trappability	Horn growth
	Estimate (CI_95%_)	Estimate (CI_95%_)
Trappability	–	0.76 (0.51–0.93)
Horn Growth	0.76 (0.51–0.93)	–
Survival	0.71 (0.43–0.92)	0.63 (0.36–0.85)
Availability	0.20 (−0.28–0.64)	0.57 (0.14–0.89)
Fate	−0.07 (−0.52–0.41)	0.25 (−0.22–0.70)

*Note*: Mean estimates of the posterior distributions are presented with their 95% credibility intervals.

## 4 DISCUSSION

We found consistent individual differences in behaviour and growth in a harvested wild terrestrial population indicating that, as proposed in fisheries (Arlinghaus et al., 2017; Diaz Pauli & Sih, 2017), hunting could act indirectly as a selective force on behavioural traits (Leclerc et al., 2017). We also found that trappability was associated with greater horn growth from ages 1 to 4, a period of major horn growth in that species (Bonenfant et al., 2009), supporting the contention that these traits are part of an integrated phenotype (Biro & Stamps, 2008). Similar results were reported in aquatic systems where higher risk acceptance was associated with faster growth (e.g. Biro et al., 2014). Previous research on associations between life‐history and behavioural traits was, however, often done in laboratories or under controlled conditions (Biro & Stamps, 2008). It was suggested that such associations might not be as straightforward in complex environments (Adriaenssens & Johnsson, 2009). A meta‐analysis found no clear direction for behaviour and life‐history traits correlations (Royauté et al., 2018) and studies in the wild have shown a range of results from no correlation (Haines et al., 2020), to positive and negative correlations (Réale et al., 2000) or context‐dependent correlations (Vetter et al., 2016). Our results suggest that a positive correlation between risk‐taking behaviour and growth can occur in the wild.

Trappability and horn growth were also both correlated with early‐life survival. More risk‐prone and faster growing individuals showed higher survival when there was no hunting mortality. The Ram Mountain bighorn sheep population is isolated with relatively low levels of predation except for sporadic episodes of high predation by specialist cougars (see Réale & Festa‐Bianchet, 2003). In this context, taking more risk might improve resource acquisition without involving a higher predation risk. Furthermore, early‐life mortality occurs mostly during winter months when food resources are limited. Natural selection in early‐life may thus favour more risk‐prone and faster growing individuals that acquire more resources and are in a better condition during winter months, leading to higher survival. This positive effect could mask the expected growth‐mortality trade‐off (Réale et al., 2010; Stamps, 2007). A recent meta‐analysis also found that risky behavioural phenotypes live longer in the wild (Moiron et al., 2020). These authors suggested that these individuals might be of overall higher quality, resulting in higher survival.

For rams that survived to at least 6 years of age, early‐life horn growth was also positively correlated to availability to hunters and probability of being shot at or prior to age 6. Thus, individuals who possessed favourable traits in early life were also those more likely to be killed by hunters at a young age, limiting their reproductive opportunities. This fits a paradox previously described in this system by Bonenfant et al. (2009), where natural and artificial selective pressures act in opposite directions. Faster growing individuals were favoured by natural selection through higher survival at young ages, but the fastest growing among them were also more vulnerable to artificial selection through their availability to hunters and probability of being shot before they could reproduce. This led to hunting‐induced selection on horn length (Coltman et al., 2003; Pigeon et al., 2016), opposed to early‐life natural selection.

We found no correlation between early‐life trappability and vulnerability to hunters among rams that survived early life. Previous studies that found hunting‐induced selection on behavioural traits in wild terrestrial populations (Ciuti et al., 2012; Leclerc et al., 2019; Lone et al., 2015) used direct measures of individual activity and space use during the hunting season. We used a measure of overall risk taking in early life, a few years before they could be shot for most individuals. Even if males showed consistent individual differences in overall risk taking in early life, it is possible their behaviour might change later in life (Ciuti et al., 2012).

**FIGURE A3:**
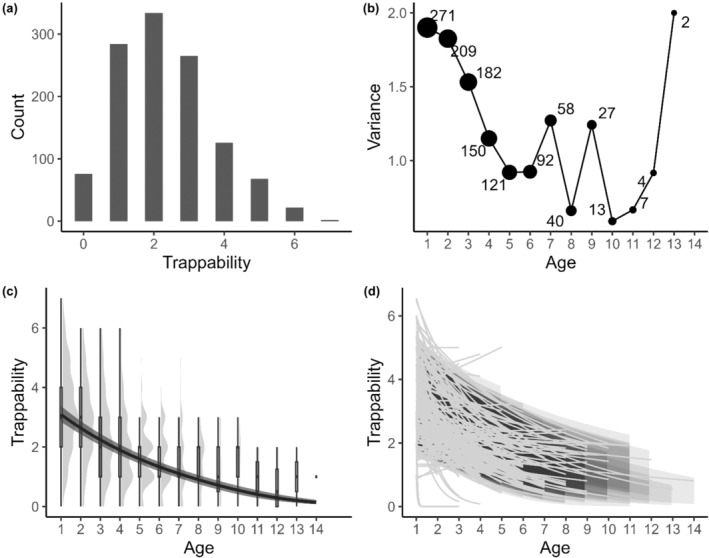
Variability of trappability with age of male bighorn sheep at Ram Mountain, Alberta, Canada. (a) Distribution of trappability for all ages. (b) Variance in trappability per age. The size of the dots and the number next to them reflect the sample size used to calculate the variance, that is, the number of individuals of that age. (c) Population‐level effect of age on trappability. The line represents the model's predicted values of trappability with age, at mean population size. The shaded area around the line represents the 95% uncertainty interval of the predicted response. The distribution of the raw data is represented in the background. (d) Individual trajectories of trappability with age. Grey lines represent individual Poisson regressions of trappability as a function of age using the raw data. The shaded area represents the overlapping 95% prediction intervals of the model for each individual accounting for population size and random intercepts and slopes of individual identity.

Post hoc analyses of ram trappability over their entire life show a decrease in mean trappability and in the among‐individual variability in trappability with age (see Appendix 2: Figure A3). Among‐individual differences in risk‐taking behaviour in early life disappear over time. These differences impact growth and survival in early life but do not appear to be important later in life. This effect could be explained by the social structure of bighorn sheep. In early life, rams are for most of the year in nursery groups with adult females and lambs, while older males (4+ years old) form bachelor groups consisting of only adult males (Festa‐Bianchet, 1991; Ruckstuhl, 1998). In our study system, nursery groups are usually larger and intraspecific competition might be greater compared to bachelor groups which are small and where intraspecific competition might be low. Population density, for example, does not affect horn growth of males after 4 years old (Jorgenson et al., 1998). A more risk‐prone behaviour might therefore be associated with more resource acquisition early in life as suggested by Biro & Stamps (2008), but not as much after 4 years old, resulting in a weaker link between trappability and horn growth among rams available to hunters. Since males are gregarious, spotting one individual might also lead to the discovery of a group of males. Hunters are then likely to shoot the largest‐horned male in a group, not the first one they spotted, possibly explaining why we found no selection on early‐life trappability through trophy hunting.

Our results illustrate that correlations between behaviour, morphological and life‐history traits can be context‐dependent and vary over ontogeny. They also highlight the difficulty in formulating general predictions about harvest‐induced selection on behaviour. Careful considerations of species ecology, harvesting regulations and harvest methods used are required before forging such predictions. Exploring causal mechanisms might also help make more accurate inferences on how behaviour affects fitness and evaluate the potential for harvest to drive selection on behaviour in wild animal populations. Most selection analyses focus on correlation between phenotypic and fitness traits and assume causality (Kruuk et al., 2003). Covariance between two or more variables, however, does not imply direct causation (Shipley, 2016) and correlations between phenotypical traits and their covariance with fitness components can arise if environmental conditions affect each trait independently or because of autocorrelation in fitness, giving biased impressions of selection (Kruuk et al., 2003; Marrot et al., 2015).

## AUTHOR CONTRIBUTIONS

**Pier‐Oliver Cusson:** Conceptualization (equal); data curation (lead); formal analysis (lead); funding acquisition (supporting); investigation (equal); methodology (equal); visualization (lead); writing – original draft (lead); writing – review and editing (lead). **Fanie Pelletier:** Conceptualization (equal); funding acquisition (lead); investigation (supporting); methodology (equal); project administration (lead); resources (lead); supervision (lead); writing – original draft (supporting); writing – review and editing (supporting).

## CONFLICT OF INTEREST STATEMENT

The authors declare no conflict of interest.

## Data Availability

Data and script are stored on Dryad Digital Repository: https://doi.org/10.5061/dryad.t4b8gtj8c.

## APPENDIX 1 AMONG‐YEAR VARIATION

**TABLE A1:** Among‐year differences in early‐life trappability and horn growth of bighorn sheep rams at Ram Mountain, Alberta, Canada.

	Trappability		Horn growth	
	Estimate (CI_95%_)		Estimate (CI_95%_)	
*Rp*	0.235	(0.137–0.368)	0.241	(0.153–0.353)
*Rp*_adj_	0.562	(0.369–0.754)	0.341	(0.228–0.473)
CV	0.172	(0.128–0.228)	0.135	(0.102–0.177)

*Note*: Estimates are the mean estimate of the posterior distributions and are accompanied by their 95% credibility intervals. *Rp* is the repeatability, *Rp*_adj_ is the adjusted repeatability (fixed effect variance removed from denominator) and CV is the coefficient of variation.

**TABLE A2:** Among‐year correlations between early‐life trappability, horn growth and survival of bighorn sheep rams at Ram Mountain, Alberta, Canada.

	Trappability		Horn growth	
	Estimate (CI_95%_)		Estimate (CI_95%_)	
Trappability	–		−0.52	(−0.77 to −0.20)
Horn Growth	−0.52	(−0.77 to −0.20)	–	
Survival	0.11	(−0.60 to 0.76)	0.35	(−0.45 to 0.87)

*Note*: Mean estimates of the posterior distributions are presented with their 95% credibility intervals.
